# Entirely Right-Sided Colon: A Case Report

**DOI:** 10.7759/cureus.99553

**Published:** 2025-12-18

**Authors:** Catherine J Llera Martin, David Howden, Zahur-Saleh Subedar, Rita Miwalian, Tanisha Shankar, Farnaz Azizi, Ronald Carlo Principe, Yosef Maher, Elizabeth Taylor, Ellen Fricano

**Affiliations:** 1 Anatomical Sciences, Western University of Health Sciences, Pomona, USA; 2 Internal Medicine, Kern Medical Hospital, Bakersfield, USA

**Keywords:** anatomical variation, cadaveric dissection, embryology, gross anatomy, malrotation of gut

## Abstract

Intestinal malrotation is a rare congenital anomaly occurring in approximately 0.2% of live births that can lead to severe complications, including midgut volvulus, short bowel syndrome, and death. This study presents a unique case of an entirely right-sided colon with associated variations in vascular structures. Possible etiologies for this anatomical anomaly are discussed, along with its clinical implications.

A detailed cadaveric dissection was performed on a donor patient at Western University of Health Sciences. The most interesting finding was that the entire colon was on the right side of the abdominal cavity, while the small intestine was on the left. The transverse colon was unusually short and constricted. The greater omentum lacked attachment to the transverse colon. The descending and sigmoid colon were supplied by the superior mesenteric artery instead of the inferior mesenteric artery. An abnormal artery branching from the inferior mesenteric artery supplied an intraperitoneal mass with the appearance of fatty tissue.

Intestinal malrotation is frequently misdiagnosed, partly because patients tend to present with nonspecific abdominal symptoms. Although this patient’s exact medical history is unknown, the family reported generalized gastrointestinal issues, which could be accounted for by the intestinal configuration observed. This unusual case highlights the importance of studies on the prevalence, variations, and clinical consequences of malrotation to improve diagnostic and management strategies.

## Introduction

Malrotation of the intestines is a congenital condition that includes partial to complete failure of the midgut to complete its full 270-degree counterclockwise rotation around the superior mesenteric artery during embryonic life [1]. Most severe cases of malrotation are discovered during infancy when complications such as midgut volvulus develop, resulting in sudden bilious vomiting [1]. Diagnosis of malrotation is rare in adulthood and includes a spectrum of presentations ranging from vague abdominal pain to bowel ischemia and obstruction [2,3]. The incidence of malrotation of the gut is reported to be about 0.2% of all live births worldwide [4]. However, it is difficult to estimate the true incidence of malrotation in adults because most patients remain asymptomatic and are not diagnosed until incidentally noted on imaging [4]. Gut malrotations can be classified through the following types: non-rotation, incomplete rotation, reverse rotation, and anomalous fixation of the mesentery [5]. Of documented cases of gut tube anomalies and malrotation, the presentation of an entirely right-sided colon has not been previously described in the literature to the authors’ knowledge.

During typical embryonic development, the midgut elongates into a primary intestinal loop, supplied by the superior mesenteric artery and its branches [1,6]. Due to rapid foregut expansion, physiological herniation occurs into the umbilical cord, where the loop undergoes a 90-degree counterclockwise rotation around the superior mesenteric artery [6]. During the third month, the intestines return to the abdominal cavity, completing an additional 180-degree counterclockwise rotation [6]. The jejunum retracts first, followed by the rest of the bowel, with the cecum initially positioned in the upper right quadrant before settling in the lower right quadrant [6]. The hindgut forms the distal third of the transverse colon, descending colon, sigmoid colon, rectum, and upper anal canal. Blood supply follows gut division: the celiac trunk supplies the foregut, the superior mesenteric artery supplies the midgut, and the inferior mesenteric artery supplies the hindgut. Blood vessels are housed within a mesentery, a peritoneal fold that suspends them within the abdominal cavity [6].

When the gut tube, neurovascular structures, or mesenteries fail to form or rotate in the predicted manner, congenital malrotations or anatomical variants may result [4-6]. One well-documented but rare anomaly occurs when the primary intestinal loop only completes a 90-degree counterclockwise rotation and fails to complete the full 270-degree rotation. This results in the colon and cecum being the first to detach from the umbilical cord, settling on the left side of the abdominal cavity, thereby creating a left-sided large intestine and a right-sided small intestine [6]. Another known gut rotation defect is when the primary intestinal loop completes only one 90-degree clockwise rotation, resulting in the transverse colon lying posterior to the superior mesenteric artery and passing posterior to the duodenum [6]. A final well-documented malrotation, often treated surgically, is Ladd’s band. This fibrous tissue band passes from a left-sided cecum to the duodenum, causing a potential site for compression and obstruction [7,8]. These “classic” presentations have been well described in the literature, and the Ladd’s procedure, which involves cutting bands of abnormal tissue and repositioning the bowel, has been a standard treatment for malrotation since it was discovered in the 1930s [8].

The purpose of this study is to present a unique anatomical variation: an entirely right-sided colon with associated arterial variations, and to discuss possible etiologies that could lead to this anomaly and its clinical implications.

## Case presentation

General description

**Figure 1:**
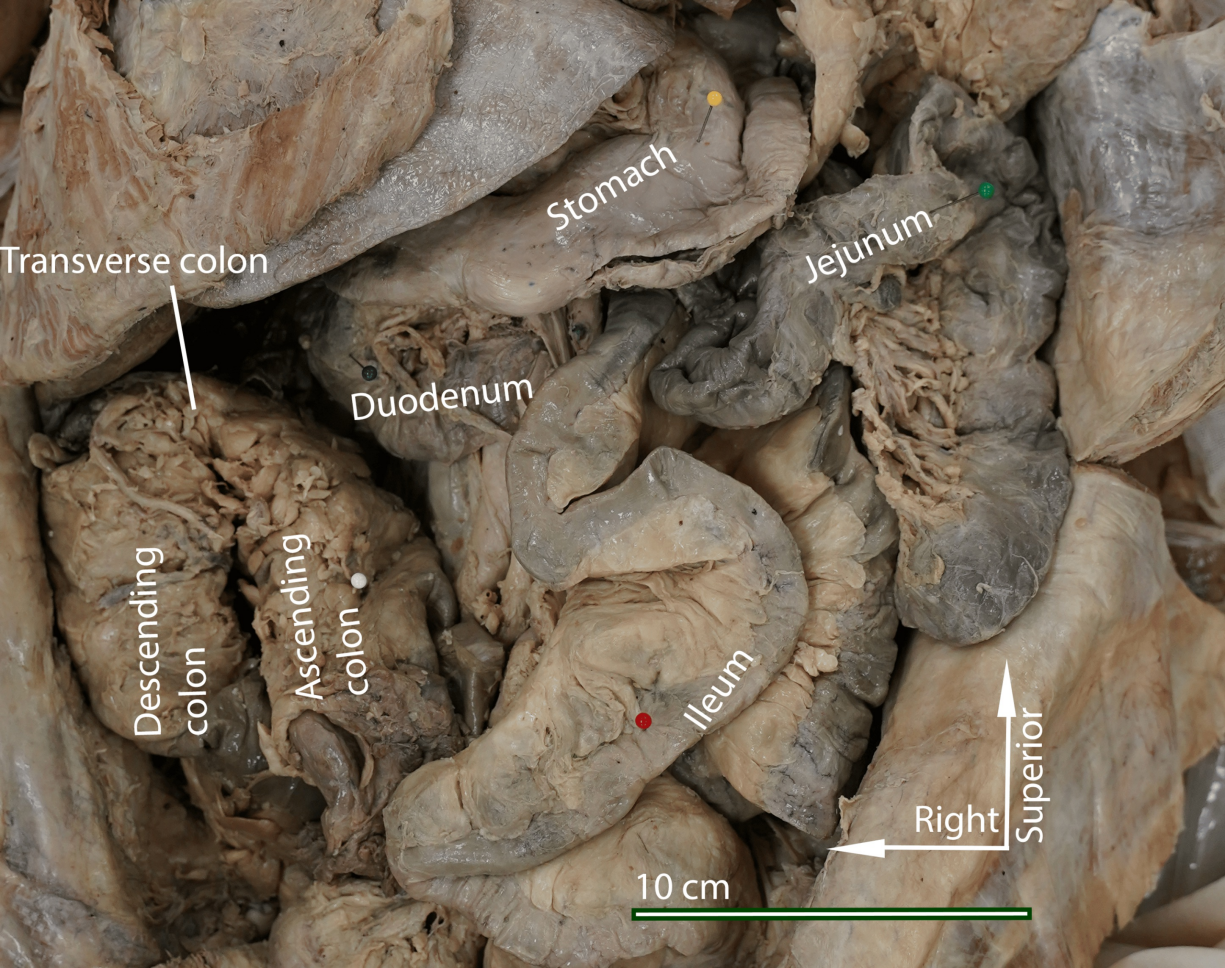
Donor patient abdominal cavity in situ Picture taken by the authors of the donor patient's abdomen with the greater omentum reflected, revealing the configuration of the intestines with the ascending colon reflected medially to show the course of the bowel. The entire colon was on the patient's right side, and the small intestine was on the patient's left. White arrows indicate standard anatomical directions.

During the routine dissection of the abdominal cavity in the body of an 85-year-old female donated cadaver, it was discovered that the colon was entirely located on the right side, with the entirety of the small intestine to the left (Figure 1). On inspection of the general abdomen, there was no evidence of abdominal surgery or intervention noted in the donor’s medical history or observed during dissection. The greater omentum was attached to the stomach and proximal part of the duodenum as expected but exhibited no attachment to the transverse colon. Instead, the greater omentum was draped anterior to the small intestines on the left side of the abdominal cavity. It was attached to the parietal peritoneum on the left body cavity.

Gastrointestinal findings

Foregut

Foregut findings did not deviate from typical gross anatomy: the third part of the duodenum traveled posterior to the superior mesenteric artery and anterior to the abdominal aorta [8]. The second and third sections of the duodenum were retroperitoneal.

Midgut

The jejunum and ileum were found entirely within the left side of the abdominal cavity. The final segment of the ileum traveled anteriorly, inferiorly, and to the right. It terminated at the ileocecal junction in the right iliac region. The cecum and ascending colon were intraperitoneal and located anterior to the descending colon on the right side.

Hindgut

**Figure 2:**
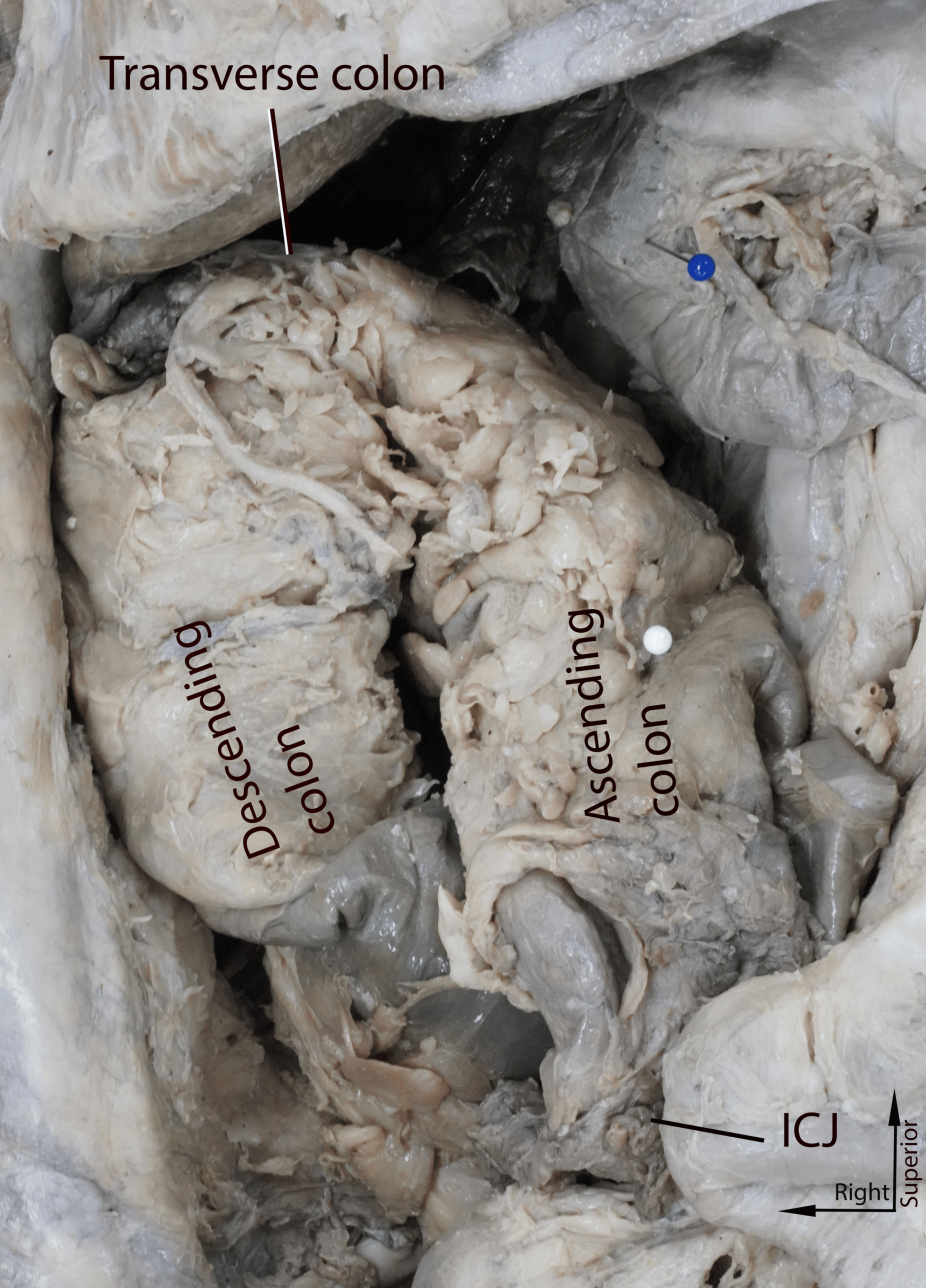
Close-up view of the colon Picture taken by the authors of the donor patient's colon displaced to the right, demonstrating the shortened transverse colon. The ascending colon, located initially anterior to the descending colon, has been shifted medially in this image to better illustrate the truncated transverse segment. Arrows indicate standard anatomical directions. ICJ: ileocecal junction

In general, the entire colon was contained in the right side of the abdomen (Figure 1). The transverse colon was unusually short and thickened. There was a single flexure midway through the transverse colon, approximated at the expected location of the hepatic flexure (Figure 2). The descending colon was retroperitoneal. A clear sigmoid colon was not identified; instead, the descending colon terminated in a straight segment that led to the rectum (Figure 2).

Vascular findings

**Figure 3:**
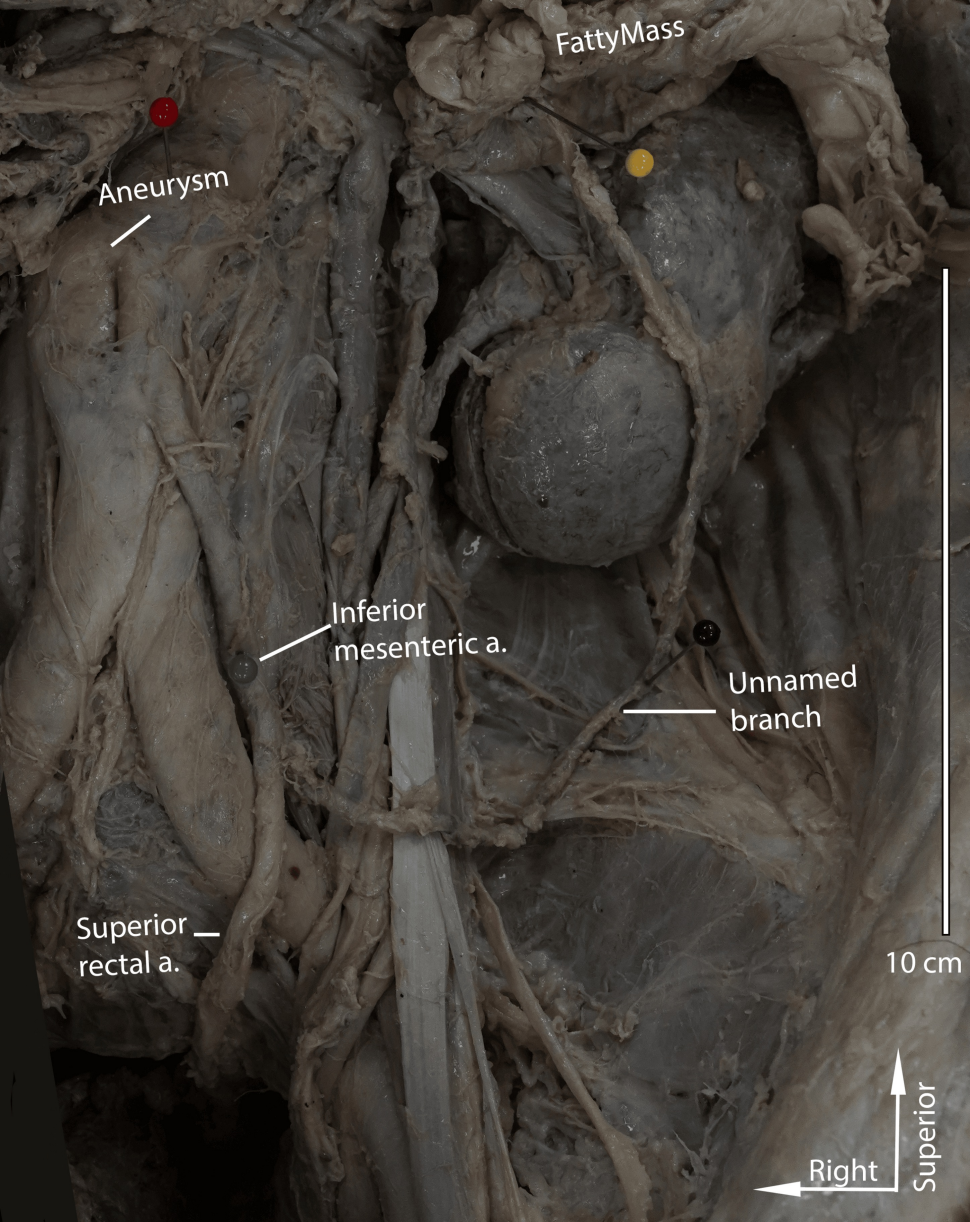
Inferior mesenteric artery and its branches Picture taken by the authors of the donor patient's inferior mesenteric artery branching off the abdominal aorta. An unnamed vessel branches from the inferior mesenteric artery and supplies an isolated fatty mass. The white arrows indicate standard anatomical directions.

**Figure 4:**
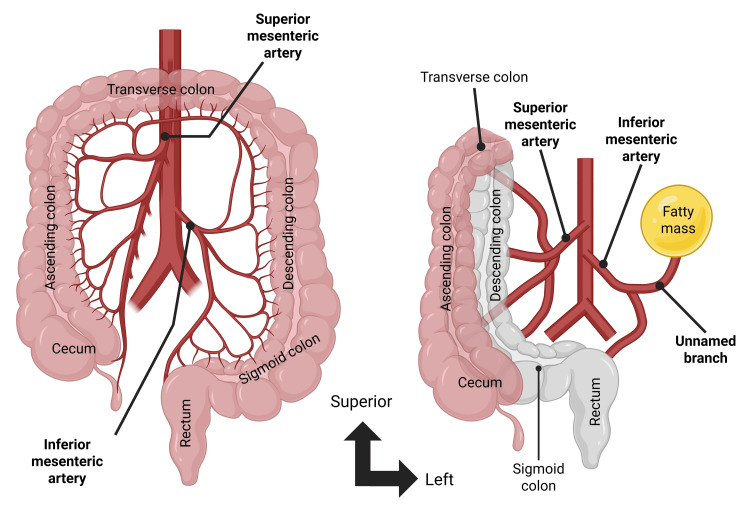
Arterial supply Diagram created by authors illustrating the most common anatomic configuration (left) and the configuration observed (right) in which the descending and sigmoid colon receive their blood supply from the superior mesenteric artery. In contrast, the inferior mesenteric artery supplies an abdominal fat pad. The arrows indicate standard anatomical directions. Created in BioRender. Llera Martin CJ (2025); https://BioRender.com/jpcx0y4

The most notable divergence from typical vascular anatomy was that the superior mesenteric artery supplied the descending and sigmoid segments of the colon. The inferior mesenteric artery had two branches: a superior rectal artery, which followed a standard course, and a second unnamed branch that supplied a large mesenteric fatty mass found in the upper left quadrant of the abdomen (Figures 3-4). The aorta proximal to the superior mesenteric artery was dilated, consistent with a saccular abdominal aortic aneurysm.

## Discussion

As presented above, this donor patient exhibits numerous deviations from typical gastrointestinal anatomy, most notably, a right-sided colon, a sigmoid colon supplied by the superior mesenteric artery, and a greater omentum lacking attachment to the transverse colon. The atypical arrangement of the midgut, hindgut, and associated structures in this cadaver raises questions about the specific deviations from normal embryological development that could explain these findings. A corresponding description of the observed malrotation could not be found in the literature [3,9-11]. Furthermore, while numerous reports describe malrotation, the mechanisms and genetic factors underlying malrotation or its absence remain poorly understood [12].

In the absence of directly comparable case examples, we present possible scenarios that could have led to the observed malformations, based on current understanding of gut malrotations. One mechanism that could have produced these findings is that the initial 90-degree counterclockwise turn around the superior mesenteric artery and the subsequent 180-degree turn as the intestines returned into the peritoneal cavity occurred as expected. However, insufficient elongation of the large intestine prevented it from extending to the other side of the abdomen, resulting in an entirely right-sided colon. The co-occurrence of a shortened intestine and malrotation has been previously reported [12]. Thus, abnormal intestinal elongation may contribute to the atypical positional outcomes observed in these cases. To account for the retroperitoneal positioning of the descending colon, there may have been an additional rotational event, like an extra 90-degree counterclockwise rotation after the 180-degree turn as the abdominal contents returned to the peritoneal cavity. This extra 90-degree turn would also push the small intestine towards the right side of the abdominal cavity. Still, because the colon was already there, the small intestine was forced to settle into the left side of the peritoneal cavity. The second mechanism by which the observed malformations may have occurred is that, during the return of the abdominal contents to the peritoneal cavity, an additional rotation occurred around the superior mesenteric artery, resulting in a 90-degree counterclockwise rotation of the midgut. Specifically, the large intestine underwent a clockwise rotation of approximately 180 degrees around the craniocaudal axis. This abnormal rotational movement would have repositioned the descending colon posteriorly, resulting in its retroperitoneal fixation.

While this patient’s medical history is unknown, except for family reports of vague gastrointestinal complaints, the shortened length of the transverse colon may have presented a site for potential obstruction and decreased motility. Compared to manifestations of malrotation that present in infancy, such as bilious vomiting, abdominal distention, and failure to thrive, most adults who present with vague abdominal complaints are misdiagnosed with conditions such as inflammatory bowel disease, peptic ulcer disease, and gastroesophageal reflux disease [13-15]. Malrotation in adults may present in acute manifestations such as a midgut volvulus or a chronic and indolent course, which may present with years of vague gastrointestinal symptoms such as constipation, abdominal pain, and emesis [16]. In patients with no prior abdominal surgery, gut malrotation should remain on the broad differential diagnosis list [16]. Contrast-enhanced CT remains the primary and most effective diagnostic tool for visualizing and diagnosing intestinal malrotations [3]. The patient in this case had no evidence of prior abdominal surgery or volvulus, so it is unlikely that any of these serious complications occurred.

Limitations

Several limitations of this study are essential to acknowledge. First, this is a single cadaveric case, which limits its generalizability. Second, limited health information was available about the individual, so we are unable to comment on how the anatomical variation affected their life and overall health. Finally, while several explanations for the anomaly are presented, we are unable to draw causal inferences because the literature directly testing how and why malrotations occur is scarce. This is an avenue of research that should be further explored.

## Conclusions

Given the unusual anatomical findings in this case, it is essential to recognize that embryological anomalies such as malrotation can present in subtle and variable ways, often eluding diagnosis until adulthood or remaining undiagnosed altogether. Our observations emphasize the critical need for heightened clinical awareness of intestinal malrotation and its variants, especially in adults presenting with nonspecific gastrointestinal symptoms. Early recognition and appropriate imaging are essential to prevent potential complications such as volvulus and bowel ischemia. Further investigation into the spectrum of rotational anomalies and their clinical implications may improve our understanding of these conditions and aid in timely diagnosis and management.

## Appendices

The donor referenced in this study was obtained through the Human Body Donation Program, an anatomical donation program, at Western University of Health Sciences. The patient gave informed consent for their remains to be used in medical education and research. The dissection of the donor body and the discovery of anatomical variations were conducted in accordance with established university protocol. All authors completed a thorough, detailed dissection of the donor patient. The specific dissection described in this article was done with an emphasis on the midgut and hindgut. Neurovascular abnormalities and the positioning of other gut structures were noted and documented with photography during dissection.
